# Yellow papules: the iceberg of systemic elastorrhexis

**DOI:** 10.11604/pamj.2013.14.106.1225

**Published:** 2013-03-17

**Authors:** Asmae Elhatimi, Amal El Bakkal, Soraya Iguermia, Maryame Meziane, Ouafae Mikou, Fatima Zahra Mernissi

**Affiliations:** 1 Department of dermatology, HassanII university hospital center, Fez, Morocco

**Keywords:** Systemic elastorrhexis, pseudoxanthoma elasticum, Gronblad-Strandberg-Touraine

## Abstract

Systemic elastorrhexis is a multisystem genetic disorder characterised by dystrophic mineralization of soft connective tissues in a number of organs, including the skin, the eyes and the arterial blood vessels. Although the eye and skin findings have for years attracted the attention of ophthalmologists and dermatologists, the systemic nature of the disorder has not received sufficient attention among internists and many patients with this disorder have undoubtedly been unrecognized. We reported a case of systemic elastorrhexis redressing the diagnosis of vascular leucoencephalopathy of an unknown aetiology for many years.

## Introduction

Systemic elastorrhexis, Gronblad-Strandberg-Touraine syndrome or pseudoxanthoma elasticum is a rare systemic inherited autosomal recessive disease, causing progressive mineralization and fragmentation of the elastic fibres, characterized by its clinical heterogeneity and various affected tissues, the most affected being cutaneous, ocular, and arterial [1].

## Patient and observation

A 58 year-old-man, with past family history of consanguinity, hypertension and a personal history of idiopathic hypertension for 10 years was admitted at the department of neurology, Hassan II University Hospital Center for status epilepticus with demential and tetrapyramidal syndromes. The ophtalmological examination showed a macular degeneration and the cardiovascular exploration found mitral, aortic and tricuspid insufficiency. Resonance magnetic imaging of the brain revealed a vascular leucoencephalopathy with numerous supraprotuberantial lesions of low intense signal T1 and hypersignal T2 and flair. The chest X-ray was normal. Laboratory data including blood count, erythrocyte sedimentation ratio, thyroid stimulating hormone, hepatitis, treponema and HIV blood serologies were normal.

**Figure 1:**
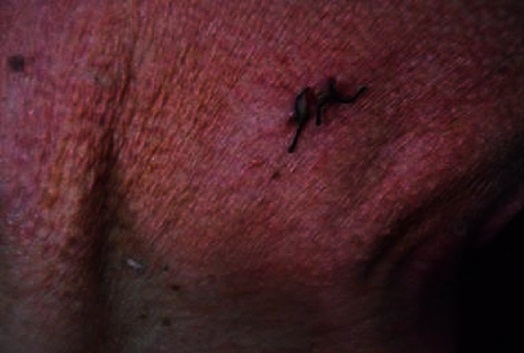
Multiple small yellowish papules organized in reticulated pattern in the lateral side of the neck

**Figure 2:**
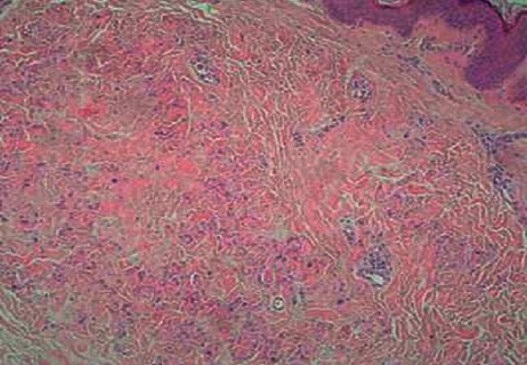
Presence in the mid dermis of degenerated and fragmented elastic fibers with a granular appearance (HESX20)

The cytologic, bacteriologic, treponema serology and cerebrospinal fluid pressure were also normal. During his hospitalization a dermatological exam for lesions in the patient's neck was required and found multiples small yellowish papules with size ranging from 1 to 5 mm, organized in reticulated pattern on the posterolateral side of the neck (Figure 1). The age of their appearance wasn't precise. The diagnostic of systemic elastorrhexis was suspected and confirmed by the biopsy of the affected skin showing in the mid-dermis a degenerated, fragmented and irregularly clumped elastic fibers of a granular appearance (Figure 2). The patient was treated symptomatically for his neurological and cardiovascular complications by Phenobarbital, platelet inhibitors, statine and by angiotensin-converting enzyme inhibitor. The genetic study was not done because of the lack of molecular biology's specialized laboratory in our country.

## Discussion

Systemic elastorrhexis is a rare genetic disorder, characterised by a progressive mineralization and fragmentation of the elastic fibers in the skin, eyes, and blood vessels. It was first considerided as a skin disorder in France when the first autopsy case was described in TB patients in 1884 and reported by Balze who thought the condition belong to xanthomtoses. In 1889 Chauffard was interested by another case whose biopsy specimen noted that the degenerating tissues stained like elastin, giving the disorder its present name. Thereafter this second patient developed a multiples gastrointestinal haemorrhages and amblyopia in 1903, and because of the wide interest in this case, Hallopeau and Laffite reported the ocular findings. Although no mention was made of angiodes streaks, note was taken of chorioretinitis involving the macula and the suggestion that the condition involve systemic illness was established. Contemporaneous with the discovery of pseudoxanthoma elasticum by the dermatologists, ophthalmologists took an interest in another disorder which was to be linked to the skin disease many years later it was the angiode streaks [2].

The prevalence of systemic elastorrhexis is estimated to 1/25,000 to 1/100,000 people [3], with female predominance [4]. In our observation, the patient was of male gender. It's a systemic disease with polymorphic clinical features depending on the organ affected. The skin is affected in about 70% of cases, typically as a small yellow papules in reticulated pattern resembling plucked chicken skin in the big flexor areas, usually at the age of thirteen and the diagnosis is made generally within the nine years following the appearance of cutaneous lesions. In our patient the cutaneous lesions were characteristic of systemic elestorrhexis but they were discreet and asymptomatic; that is why the age of their appearance couldn't be determined. In some cases the cutaneous findings may be marqued and inesthetic, motivating consultation in dermatology, especially in women [1]. The light microscopy examination of the damaged skin shows, in the middle and deep dermis the accumulation of thick, fragmented and irregularly clumped elastic fibres given a granular appearance to the corium and the deposition of calcium in this degenerated material [5] as seen in the biopsy specimen of our patient. The eyes findings are also frequent, up to 86%, shortly after the appearance of the cutaneous lesions, usually between the age of 14 and 25 years. Described either as angiodes streaks, as retinal hemorrhages or as macular degeneration [1]. In our case the ocular findings were represented by a macular degeneration. The third affected system is the cardiovascular, later after the cutaneous and the ocular ones, represented by decreased peripheral pulses, hypertension and brain stroke. The impairment of this system may condition the vital and the functional prognosis as it was clearly illustrated by our patient who developed a malign hypertension with leucoencephalopathy manifested by status epilepticus, demential and tetrapyramidal syndromes making the patient bedridden and invalid. Given the multisystemic nature of the condition, other organs may be affected as the gastrointestinal and the genital systems, expressed especially by haemorrhages.

In 1992, the diagnostic criteria for systemic elastorrhexis was established to classify the condition in three categories; the major ones include characteristic skin involvement, ocular findings and histopathological features, minor criteria include familial history of the disease and calcification of elastic fibers in non-affected skin [6]. We note here that cardiovascular symptoms weren't include in these criteria which haven't been unanimous for all authors. Because of the incomplete phenotype of the condition, certain authors suggest to enlarge the diagnosis and to redefine the diagnostic criteria. In our patient the diagnosis was made on the skin lesions, the personal early history of hypertension of unknown etiology, the characteristic histopathological feature in the affected skin and on the familial history of hypertension. Although the skin lesions are initially the main concern of the patient in this condition, they are asymptomatic and mostly cosmetic issue and the morbidity is due to the extra cutaneous manifestations especially to eye and to cardiovascular system. Our patient illustrate the burden of the cardiovascular complications and the systemic nature of the disease, that hasn't received as sufficient attention as cutaneous and ocular findings among internists, explaining why many patients with this disorder have undoubtedly been unrecognized. In our case the diagnosis was made 10 years after the cardiovascular complications.

Systemic elastorrhexis is an autosomal recessive disorder, with inter and intra family phenotypic variability. It is caused by mutations in the ATP bindings cassette transporterC6 (ABCC6) encoding for multidrug resistance associated protein 6: a cellular transport protein. To date, over 300 distinct mutations have been identified. Even if the disease is recessive, carriers of single mutant allele of the ABCC6 were reported and they can rarely develop a severe ophthalmologic or cardiovascular manifestations [7]. In our patient the genetic counselling couldn't be done due to the absence of specialized laboratory on the molecular biology. There is still no universally effective treatment or cure for systemic elastorrhexis, treatment is still aimed at prevention and early detection of adverse ocular and cardiovascular complications, patients should have frequent evaluations by cardiologists and ophthalmologists and should be counselled on the reduction of cardiovascular risk factors, and on the activities that may increase the risk of bleeding. Our patient was treated by Phenobarbital for epilepsy, platelet inhibitors, angiotensin-converting enzyme inhibitor and statine for malign hypertension.

## Conclusion

Systemic elastorrhexis is an inherited disorder of connective tissue due to a mutation in gene located on chromosome 16p. The condition usually affects the skin and the ocular tissues and may have a vascular attack dominates its clinical presentation and prognosis. Therefore it's important to consider this disease in young adults with severe cardiovascular disease or hypertension without risk factors.
